# Diatoms in Focus: Chemically Doped Biosilica for Customized Nanomaterials

**DOI:** 10.1002/cplu.202400462

**Published:** 2024-10-18

**Authors:** Cesar Vicente‐Garcia, Danilo Vona, Annarita Flemma, Stefania Roberta Cicco, Gianluca Maria Farinola

**Affiliations:** ^1^ Dipartimento di Chimica Università Degli Studi di Bari “Aldo Moro” Via Orabona 4 70125 Bari Italy; ^2^ Dipartimento di Scienze del Suolo Della Pianta e Degli Alimenti Università Degli Studi di Bari “Aldo Moro” Via Amendola, 165/a 70126 Bari Italy; ^3^ CNR Istituto di Chimica dei Composti Organometallici Dipartimento di Chimica Università Degli Studi di Bari “Aldo Moro” Via Orabona 4 70125 Bari, Italy

**Keywords:** Diatom biosilica, Diatomite, Catalysis, Bioremediation, Bioenergetics

## Abstract

Diatoms are photosynthetic microalgae widely diffused around the globe and well adapted to thrive in diverse environments. Their success is closely related to the nanostructured biosilica shell (frustule) that serves as exoskeleton. Said structures have attracted great attention, thanks to their hierarchically ordered network of micro‐ and nanopores. Frustules display high specific surface, mechanical resistance and photonic properties, useful for the design of functional and complex materials, with applications including sensing, biomedicine, optoelectronics and energy storage and conversion. Current technology allows to alter the chemical composition of extracted frustules with a diverse array of elements, via chemical and biochemical strategies, without compromising their valuable morphology. We started our research on diatoms from the viewpoint of material scientists, envisaging the possibilities of these nanostructured silica shells as a general platform to obtain functional materials for several applications via chemical functionalization. Our first paper in the field was published in *ChemPlusChem* ten years ago. Ten years later, in this *Perspective*, we gather the most recent and relevant functional materials derived from diatom biosilica to show the growth and diversification that this field is currently experiencing, and the key role it will play in the near future.

## Graphical abstract

The possibility to chemically modify the biosilica of diatom microalgae has attracted a lot of interest. New strategies for chemical modification, either covalent, non‐covalent, and feeding‐based approaches to alter their shells composition are reported yearly. Their versatility in composition and function has yielded diatom biosilica the go‐to biomaterial for the development of biocompatible composite materials in fields from biomedicine to optoelectronics.

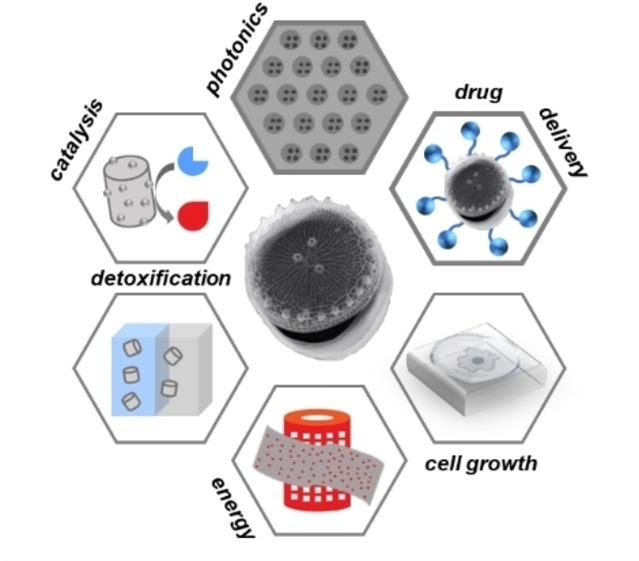

## 1 Introduction

Diatom microalgae are widely known for their unique feature of bearing an extracellular shell made of nanostructured biosilica. These photosynthetic microorganisms can be found in almost every aquatic and marine environment on Earth, and are essential to oxygen production, and to carbon and silicon marine cycles.^[1]^ Diatoms have been around for around 200 million years, in that time they have evolved into more than 100.000 species, with their corresponding unique shapes. Their beauty captivated the first microscopists in the 18th century, and now inspires the community of chemists and material scientists, from which we are part, to exploit their features for the design of novel, highly tailored materials.^[2]^

**Figure 1:**
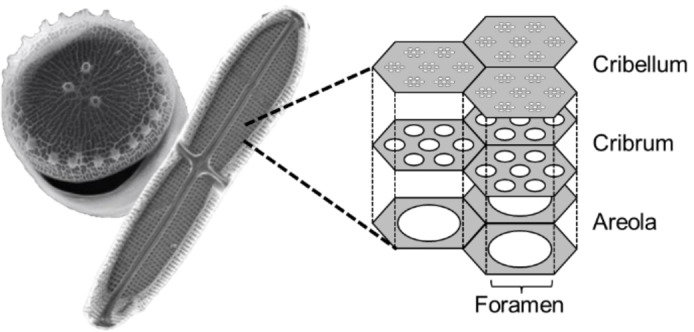
SEM images of the frustule of a centric diatom (round, with a diameter of 7 μm), a pinnate diatom (elongated, with a longitudinal size of 25 μm), and schematic representation of the micro‐/ nanostructure of their hierarchically organized pore pattern. Reproduced with permission from Ref. [22] and [54]. Copyright 2008, and 2018 Wiley.

The exceptional physical properties of diatom frustules are closely related to their biological role and biogenesis. These exoskeletons resemble a microscopic Petri dish (Figure 1), made of two valves (hypo‐ and epitheca), connected by a less‐robust band of biosilica (girdle).^[3]^ Protection from mechanical stress is presumed to be the main role of the frustule, with the complex array of pores allowing the free exchange of nutrients and waste material between the protoplasm and the environment (Figure 1). Yet, the most interesting feature of these hierarchically ordered pore patterns, is their capacity to act as natural photonic crystals. Thus, frustules can filter out harmful ultraviolet light, while concentrating beneficial photosynthetically active radiation.^[4]^ The elevated cost and difficulty of reproducing frustules’ pore size distribution and homogeneity, has made them appealing ready‐to‐use nanostructured materials.

Diatom biosilica can be gathered from freshly cultured diatoms, yielding a highly monodispersed and intact nanostructured material comprised of pristine frustules (PF), or from the fossilized deposits of expired diatoms, known as diatomaceous earth (DE) or diatomite, constituted of a heterogeneous mixture of frustule fragments from different species.^[5]^ The most appealing aspect of diatoms in nanotechnology lies in the great variety of chemical methodologies that can be combined or superimposed, including covalent, non‐covalent and metabolic‐insertion approaches, offering a colourful map of chemically decorated, nanostructured materials, which applications in biomedicine, sensing, drug delivery, energy conversion and storage, and optoelectronics have recently been reviewed.[^6^, ^7^]

Our first publication on diatom biosilica frustules as starting material to produce functional hybrid nanostructures appeared in *ChemPlusChem* ten years ago. After a decade of intense work in the field, our research group has grown both in number and reach, expanding into new and exciting uses of functionalized biosilica for many different applications. In this *Perspective*, we focus on the methodologies of diatom frustule chemical modification, including the *in vitro* approaches that use DE and PF as inorganic raw material, and those which are based on *in vivo* feeding active compounds. Taking advantage on our expertise, and with special focus on our contributions to the growth of this research field, herein we aim to set a check point on the limits and possibilities of different strategies, highlighting the most innovative and promising applications of biosilica‐derived materials.

## 2 The Biosilica Source Determines the Chemical Modification Methods

Diatom biosilica can be harvested from two main sources. DE consist of the fossilized remains of ancient diatoms that have accumulated over millions of years on lake and marine sediments. Nowadays, DE is regularly mined from sedimentary deposits and is commercially available. Alternatively, frustules can be freshly harvested from living diatom cultures, by removing the cell protoplasm via chemical or physical methods. There are several protocols available in literature to obtain PF, which harshness determines the final content of organic compounds and the reactivity of the material.[^8^, ^9^]

**Figure 2:**
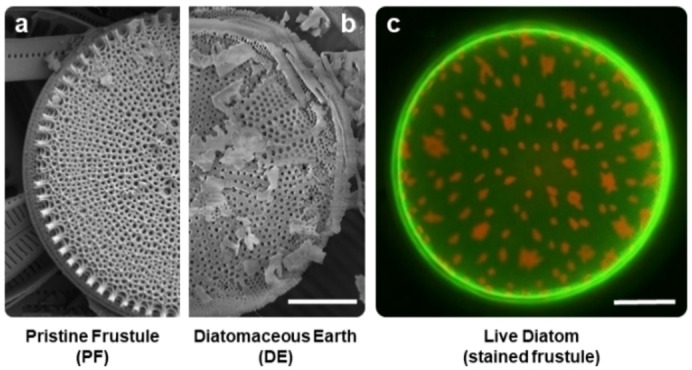
SEM images from (a) a diatom frustule, (b) a fractured frustule from a DE sample. (C) Confocal fluorescence image of a live centric diatom; chloroplasts appear in red due to natural chlorophyll fluorescence, and the silica shell in bright green from staining with Rhodamine 19. Scale bar corresponds to 5 μm for (a) and (b), and to 50 μm for (c). Reproduced with permission from Ref. [15] and [66]. Copyright 2012 Royal Society. Copyright 2024 American Chemical Society.

Even though the main component of both DE and PF is SiO_2_, there are key differences in their properties, essential to any chemical modification, and subsequently to the field of application. Biosilica frustules extracted from living diatom cultures can yield nanostructured shells with up to 97 % pure SiO_2_, after conventional cleaning.^[8]^ This kind of treatment removes mainly the organic fraction of the diatom, while preserving the intrinsic micro‐ and nanostructure features of frustules, and therefore their morphological and photonic properties. Biomass generated inside a monoculture (containing a single species of diatom), allows to obtain a homogeneous material made of exact copies of the same three‐dimensional architecture (Figure 2a). Therefore, intact shells can be used for photonic applications, i. e. as bio‐based UV‐filters and microlenses.^[10]^ The lack of impurities and preserved architecture make PF also a suitable material for biomedical applications like drug delivery,^[11]^ cell adhesion, and cell capture from biological fluids.^[12]^

On the other hand, biosilica shells from DE have been subjected to a fossilization process over a long period of time that can alter their composition, introducing Mg, Ca, Al or Fe atoms that diffuse from surrounding minerals.^[5]^ Moreover, geological shear and compression forces that act upon the fossilization process frequently result in frustules being broken into smaller particles, compromising the photonic structures (Figure 2b).^[5]^ DE commonly consists on an heterogeneous mixture of numerous species, which composition depends on the geographical location and origin period of the deposit. However, such naturally doped biosilica from DE can be useful for applications that can exploit specific properties that these metallic cations give to DE; remarkably, for their catalytic activity (i. e. for Knoevenagel Condensation),^[13]^ or for the preparation of thermoelectric materials.^[14]^ Living diatoms exhibit intact frustule architecture surrounding the cell protoplasm, as can be seen under fluorescence microscopy when stained properly (Figure 2c).^[15]^

**Figure 3:**
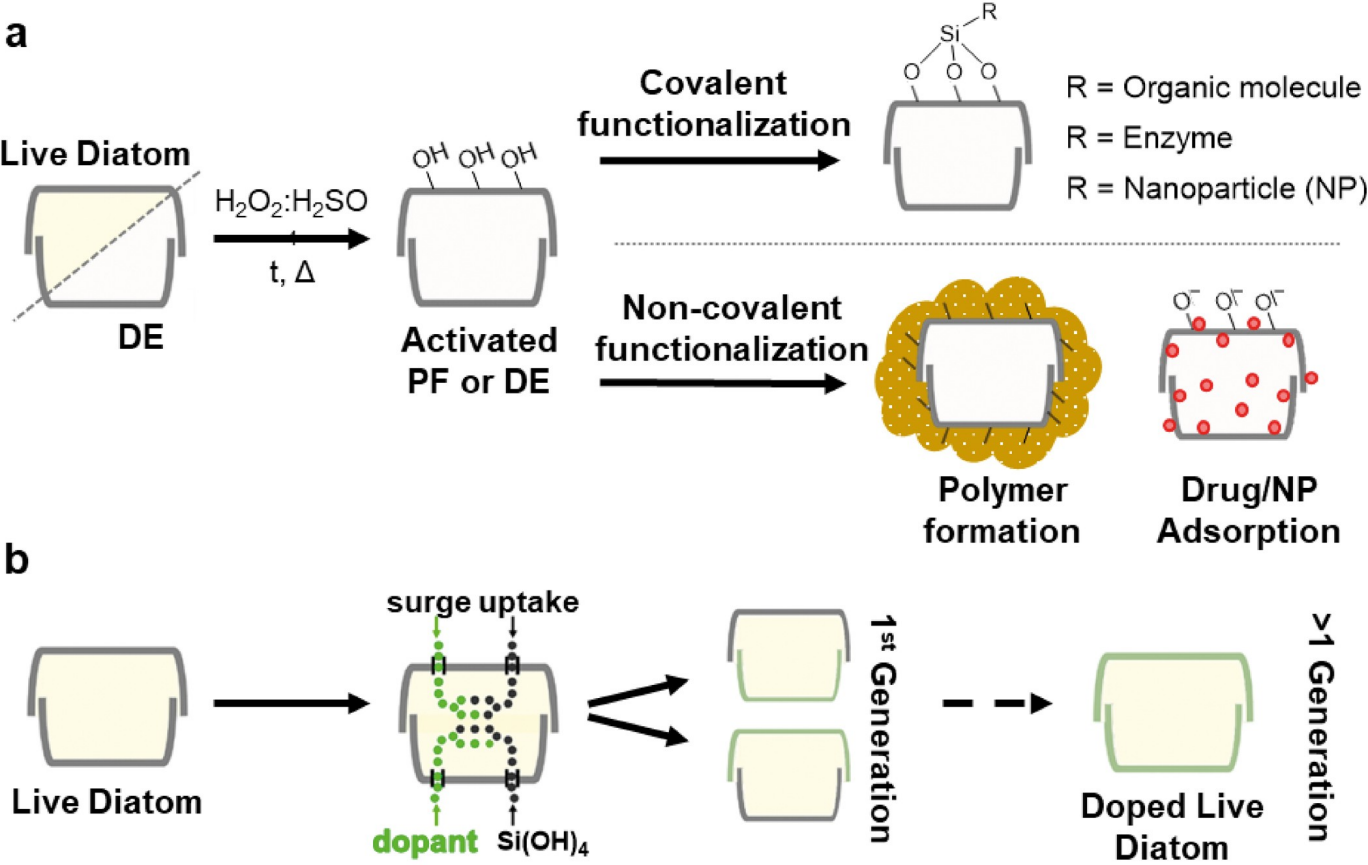
General strategies for biosilica modification. (a) Activation of biosilica via acid/oxidative treatment, followed by covalent or non‐covalent functionalization of the frustule. (b) Incorporation of dopant elements into newly generated frustules by metabolic insertion through live feeding. Reproduced with permission from Ref. [24]. Copyright 2020 MDPI.

The main strategies of chemical functionalization that are used for DE can be also applied to PF, once all the organic matter has been removed. Covalent functionalization generally provides a stronger and more durable binding of molecules to the frustule's surface. Using this strategy can also afford the integration of large elements that would otherwise be prone to be washed away due to the steric hindrance (Figure 3a). A very interesting example are biomacromolecules, such as enzymes, antibodies or even nucleic acids, that are useful in the design of hybrid nanostructures for catalysis and biosensing.^[6]^ This strategy is predominantly based on silanol condensation at the biosilica surface as first step. Bridge molecules like (3‐aminopropyl)triethoxy and (3‐mercaptopropyl)triethoxy silane (APTES and MPTES, respectively), are used to create a stable external layer over biosilica, which amino or mercapto groups can be then used for further covalent functionalization.

A clever example of this is represented by using APTES to covalently bind vitamin B12 (cyanocobalamin) to DE particles, previously activated with H_2_SO_4_ treatment, to improve selectivity in drug delivery.^[16]^ A new innovative approach was recently reported, based on the covalent binding of triethoxy(octyl)silane molecules to the surface of DE, generating biosilica with a marked apolar character, improving pharmacokinetic delivery parameters.^[17]^

Covalent chemistry can also be exploited to anchor inorganic elements, for instance, in the fabrication of versatile hybrid metal‐ceramic composites. In a different case, the generation of a layer of silica xerogel with thiol groups around the DE surface, afforded a readily active surface for the *in‐situ* formation of Au or Ag nanoparticles (NPs).^[18]^

Conversely, non‐covalent methods can be utilized to generate soft functional materials, based on non‐covalent interactions. Some of the most frequently used approaches include the adsorption of molecules into the pore network of biosilica (chemisorption and physisorption) (Figure 3a), atomic layer and chemical‐bath deposition, and thermal annealing.^[6]^ The use of bio‐inspired polymeric materials to coat DE and PF has been recently growing, due to their general biocompatibility and adhesive properties (Figure 3a). For instance, polydopamine (PDA), a bio‐inspired, melanin‐like polymer can be *in situ* generated by spontaneous polymerization in basic aqueous solution and can be used to coat DE particles. DE/PDA composites can be used as filters.^[19]^ Another well‐known biopolymer, chitosan, was successfully used to coat DE via a solvent‐casting method, yielding a composite material suitable as bone growth scaffolding, as demonstrated with human osteoblastic cells.^[20]^ Interestingly, metals can also be used to dope biosilica through non‐covalent methods. A combination of de‐sorption and calcination methods allowed to coat DE particles with Nickel atoms.^[21]^

Lastly, the major advantage of using PF instead of DE involves the possibility of inducing the *in vivo* incorporation of elements into the incipient biosilica during diatom mitotic growth. This methodology consists of providing alternative building blocks to diatoms, with similar chemical properties to silicic acid (their natural silicon source). In this way it is possible to effectively trick diatoms into generating naturally doped micro‐structured biosilica.^[6]^ The first example that evidenced this principle comprised the addition of Ge(OH)_4_ into the diatoms culture; given the similar properties of Ge(OH)_4_ and Si(OH)_4_, Ge ions were incorporated into the frustule's matrix, transforming it from a natural isolating to a semiconductor material.^[22]^ Since then, other metallic cations have been proven to be actively incorporated by living diatoms into their frustules, like Zn, Ti, Al, and Fe.[^23^, ^24^]

In a striking turn, organoalkoxysilanes (OAS) have also been reported to work as building blocks for diatom frustule biogenesis, opening the door to naturally doped biosilica with organic moieties.^[6]^ In this manner, small, tailored organic molecules can be incorporated into the biosilica matrix of the frustule. Very interesting examples include the introduction of a thiol moiety into diatom frustules, that could be later utilized for further *in vivo* functionalization;^[25]^ or more complex compounds such as fluorescent organic dyes, that can be used in photonic and optoelectronic applications, such as light filtering and trapping for energy harvesting, or biosensing.^[26]^ Other examples have demonstrated the incorporation of non‐OAS small molecules into the growing biosilica network. A beautiful example is represented by the incorporation of an iridium metal complex into living diatoms, providing highly emitting biosilica with potential use in imaging and biomedicine applications.^[27]^

Even though the incorporation mechanism is not well known in the case of non‐OAS organic molecules, the possibility of introducing such a wide range of functional elements into living diatoms makes *in vivo* incorporation a valuable and versatile approach.

## 3 Diatom Biosilica, a Multifaceted Bio‐Based Material, Suitable for Applications from Biomedicine to Electrochemistry

### 3.1 Diatom Biosilica for Bone Tissue‐Engineering and Other Biomedical Applications

In the last decade, the number of novel applications involving the use of diatom frustules as raw material has grown significantly. The interest to thoroughly characterize and understand their unique structures, has been fuelled by the motivation to exploit that knowledge into designing new smart materials.^[28]^ The routes to meticulously incorporate chemical modifications, or to introduce novel molecules to either enhance a biological function or to confer new ones, without the need for genetic modification, have greatly diversified.[^6^, ^29^] One of the first fields of application that involved the use of diatom frustules has been the fabrication of biocompatible scaffolding for bone growth and regeneration.^[30]^

**Figure 4:**
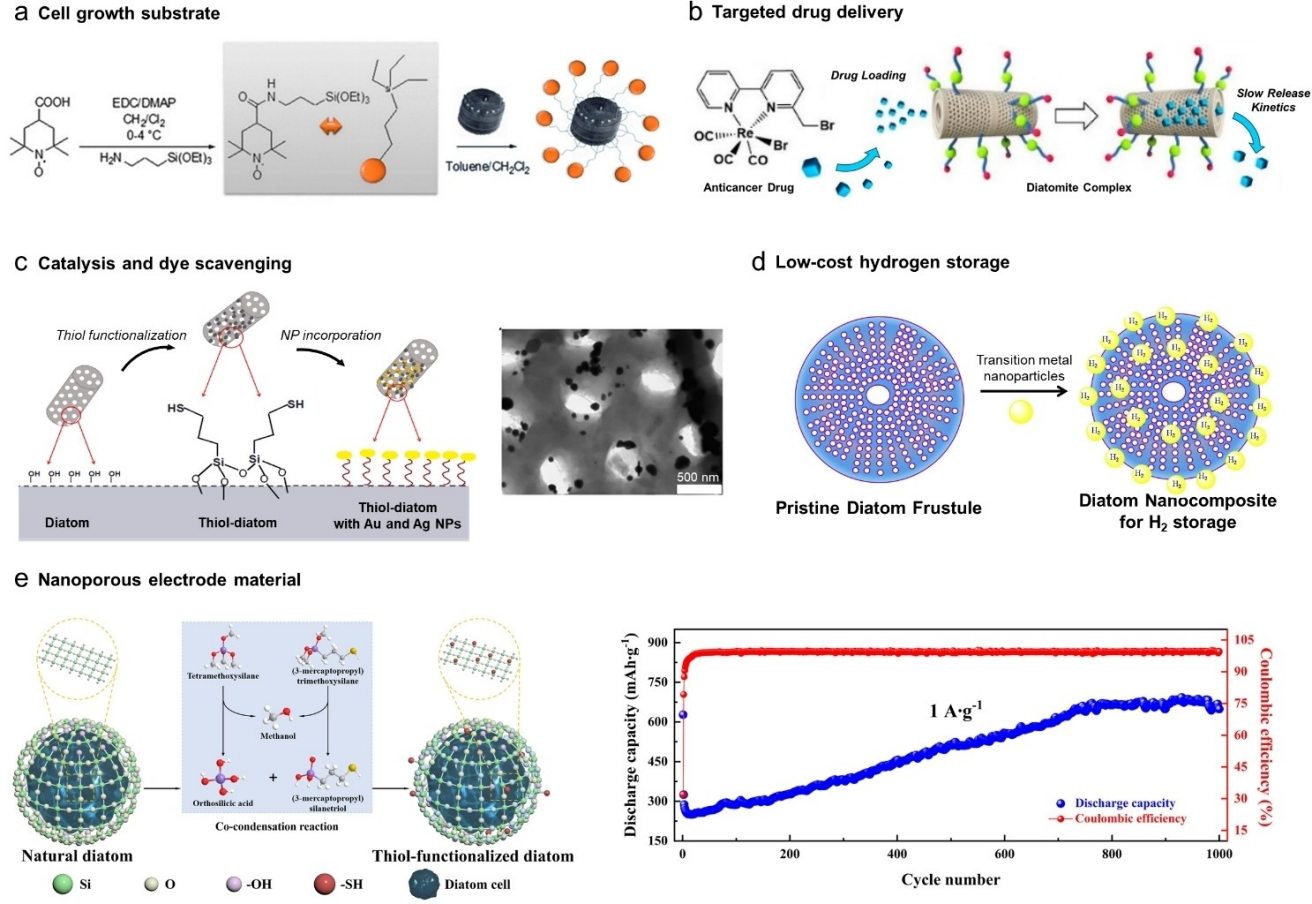
(a) Functionalization of diatom frustules with TEMPO (EDC, 1‐Ethyl‐3‐(3‐dimethylaminopropyl)carbodiimide; DMAP, 4‐Dimethylaminopyridine). (b) Schematic representation of the drug loading and slow‐release kinetics of a diatom composite. (c) *In situ* functionalization of diatom biosilica with metal nanoparticles (NPs). (d) Insertion of transition metal NPs allows to store H_2_ onto diatom biosilica. (e) Synthesis of thiol‐functionalized diatom frustules using silica colloidal network, and long‐term cycle performance of biosilica‐based battery composite material. Reproduced with permission from Ref. [18,30,39,63] and [65]. Copyright 2022 Springer Nature. Copyright 2015 Wiley. Copyright 2020 MDPI. Copyright 2022, and 2024 Elsevier.

In our first paper, PF from *Thalassiosira weissflogii* were functionalized with a radical scavenger (TEMPO or 2,6,6‐tetramethylpiperidine‐*N*‐oxyl) using APTES as linker and were subsequently loaded with an antibiotic (Ciprofloxacin), taking advantage of the high specific surface of frustules to adsorb molecules (Figure 4a). The resulting material was demonstrated to enhance the adhesion and proliferation of bone cell lines while providing both an antibiotic and antioxidant effect; envisaging it as potential bone filler.^[30]^

More recently, biosilica from the same diatom species has been covalently functionalized with both APTES and MPTES, to use as substrate for bone growth. The mercapto‐functionalized biosilica greatly influenced the vitality and shape factors of growing cells, showing a positive and intimate interaction between the rough biosilica surface and the cell's membrane.^[31]^ In a non‐covalent functionalization approach, diatomite was coated with chitosan, a biocompatible biopolymer extracted from crustaceans’ exoskeleton, and was used as substrate for bone tissue growth. The presence of the DE/chitosan composite favoured cell adhesion and proliferation, and even increased alkaline phosphate activity, essential factor for bone matrix growth.^[32]^ Diatomite has also been used as additive in combination with hydroxyapatite in polyurethane foam preparations, demonstrating improved physical properties like thermal stability and mechanical strength.^[33]^ Additionally, plain, sintered PF from *Cyclostephanos* sp. (subjected to high temperature and pressure), have been proved to favour proliferation and mineral deposition in pre‐osteoblast cultures without further functionalization. The resulting material was stable enough to withstand re‐sterilization (via autoclave), and further re‐use.^[34]^

The *in vivo* incorporation approach has also been exploited for this purpose. A bisphosphonate drug (Alendronate), usually used to treat metabolic bone diseases, was fed to a live culture of *T. weissflogii*., yielding alendronate‐functionalized frustules after acid/oxidative cleaning. This composite material showed osteoinductive properties, and even inhibition of osteoclast activity, suggesting a promising role for tissue engineering.^[35]^

### 3.2 Diatom Biosilica for Drug Delivery

Valuable properties like biocompatibility, high surface‐to‐volume ratio and a mesoporous structure have made diatom biosilica a natural choice for the design and development of drug delivery systems.^[36]^ Diatom biosilica (either DE or PF), is readily available and cheap, and does not involve the high energy demand and processing that artifitial mesoporous silica requires. There are currently numerous strategies being developed to improve biophysical properties like drug loading and release efficiency and site‐specific binding capacity, and hence adjust their features as drug delivery agents.^[37]^

When using bare diatomite as carrier for an adsorbed drug (Ophiobolin, an anticancer drug), it has been shown that a previous activation of the biosilica (acid/oxidative treatment under relatively high temperature, 80 °C) to expose a higher number of silanol groups, can enhance the drug loading and extend the drug release period.^[38]^ These relevant parameters can be further improved, by fine‐tuning the chemistry of the DE's surface. By coating DE with a highly hydrophobic OAS, triethoxy(octyl)silane, its typically more polar surface was transformed into a hydrophobic environment. This material displayed a stronger adhesion to a reconstructed human epidermis model (Epiderm^TM^, EPI‐200), and a slower release of a poorly water‐soluble drug (Naproxen), than the naked DE.^[17]^

Site‐specificity of drug delivery using a biosilica‐based system can be improved by grafting specific molecules on its surface. A clever example consisted of coating DE's surface with B12 vitamin, which is eagerly required by fast‐growing cells (such as tumor cells), to increase the delivery rate of an adsorbed drug to cancerous cells (Figure 4b).^[16]^ In a follow‐up publication, a photoactivatable element was added to the DE's surface functionalization, resulting in toxic singlet oxygen production, and consequently a 2‐fold increase in its cytotoxic efficacy.^[39]^

Diatom biosilica has been widely reported as suitable scaffold substrate as an active agent for several biomedical applications.[^11^, ^29^] Chitosan‐coated DE functioned as a safe and effective haemostatic agent.^[40]^ Different nanocomposite materials have been designed to exhibit antimicrobial activity, like PF/PDA doped with Ag nanoparticles (AgNPs),^[41]^ and DE functionalized with both TiO_2_ and AgNPs, with remarkable activity even against drug‐resistant microorganisms.^[42]^ In a similar approach, an increase in the intrinsic photoluminescence of PF upon immunocomplex formation with its complementary antigen was used as detection signal for the development of immune‐detection platforms. In one case, the solid support for immunoglobulin anchoring consisted of PF/PDA doped with AuNPs,^[43]^ and in another case, metabolically doped PF/GeO.^[44]^

### 3.3 Diatom Biosilica for Environmental Remediation

Some of the intrinsic characteristics of diatom frustules, including exhibiting a hierarchically ordered network of micro‐ and nanopores, well defined and homogeneous for every species, and an overall high surface‐to‐volume ratio, easily explain why they have been traditionally used as filtration material. Even though some of this characteristic can be reproduced in artifitial mesoporous silica, such alternatives are more expensive and difficult to create, and usually lack pore size homogeneity.^[5]^ However, their natural capacity to filter out contaminants in aqueous media is limited, hence, to use them as active part of a detoxifying system, additional elements have to be introduced. Two main strategies have been followed: (i) coating the frustule's surface with a material able to interact with and accumulate the target contaminant, with the consequent need to recover and further treat the loaded biosilica; and (ii) to include catalysts able to transform contaminants into less harmful compounds *in situ*.^[45]^

A straightforward approach consists of coating DE with PDA, which takes place in a 1‐step reaction, via the spontaneous polymerization of dopamine in mild basic solution. PDA exhibits excellent adhesive and entrapping properties, and has been demonstrated to effectively sequester low‐polar phenolic compounds from an aqueous solution;^[46]^ and uremic toxins from human serum.^[19]^ A successive step comprised the addition of an oxidative enzyme (laccase) to the DE/PDA composite, allowing to degrade some model pollutants without significantly hampering the enzyme's activity.^[47]^ Coating living diatoms with PDA can confer them remarkable resistance to external aggressors,^[48]^ and the capacity to efficiently accumulate organic pollutants.^[49]^ Additionally, functionalization of living *Phaeodactylum tricornutum* with boronic acid moieties favours biofilm adhesion to substrates and enhances their capacity to sequestrate polluting metal cations (Ar, Cr, Pb, Sb, etc) from water, entailing a promising self‐sustained living platform for bioremediation.^[50]^

A remarkable covalent approach comprised the generation of a layer of silica xerogel with thiol groups around the DE surface (via the combination of triethoxysilane and MPTES), afforded a readily active surface for the *in situ* formation of Au or Ag NPs (Figure 4c). The resulting composites demonstrated remarkable abilities for D‐glucose catalytic oxidation (relevant step in pharmaceutical industry), and for the catalytic degradation of a model dye contaminant.^[18]^ The combination of TiO_2_/DE yields very interesting photocatalytic detoxifying composites, for instance for the abatement of acetaldehyde from air,^[51]^ or for the degradation of a model dye contaminant (methylene blue) under UV light, when doped with Ytterbium.^[52]^

### 3.4 Diatom Biosilica for Photonics, Optoelectronics and Energy

*In vivo* feeding of living diatoms with organic fluorophores has allowed to generate hybrid photonic structures from frustules.^[6]^ *In vivo* incorporated fluorescent dyes are embedded into the core of the biosilica frustule, homogenously dispersed throughout the biosilica matrix, stable and protected from the outside environment. After the feeding, highly fluorescent biosilica can be extracted. Several organic fluorophores have been designed and tested for diatom feeding: triethoxysilane‐functionalized (TES), π‐conjugated organic fluorophore with a thiophene‐benzothiazole‐thiophene backbone;^[53]^ TES‐phenyleneethylene fluorophore;^[54]^ and TES‐triphenylamine fluorophore, a two‐photon absorber, great for imaging, high optical sectioning of emission and high contrast with respect to endogenous fluorophores.^[55]^ Moreover, rare‐earth metallic complexes have also been fed to living diatoms, providing highly fluorescent nanostructured material, from Iridium,^[56]^ Ruthenium and Aluminium complexes.^[57]^

The metabolic insertion of Ce atoms into living diatom frustules, later inducing the formation of CeO_2_NPs. The resulting PF/CeO_2_NPs composite exhibited interesting spectral properties, an intense violet‐blue fluorescence under UV‐radiation, and up‐conversion fluorescence in the violet region under infrared illumination. These fluorescent nanostructured materials could be used in light filtering and harvesting, biosensing, imaging and biomedicine.^[58]^

A higher level of complexity can be achieved by increasing the number of components, towards more device‐like systems. For instance, a transparent, flexible polymeric film was fabricated with embedded highly fluorescent biosilica. The frustules were coated with APTMS or (3‐aminopropyl)trimethoxy silane, before the *in situ* complexation of Eu^3+^ ions, yielding a hybrid luminescent material with peak absorption in the UV, being potentially useful for UV‐protection and photovoltaic devices. Luminescent polymeric films were obtained from DE and polyethylene terephthalate,^[59]^ and from PF and polyacrylonitrile.^[60]^ Alternatively, PF with metabolically inserted MPTMS can be further doped with Ag^+^ ions to create a composite substrate with excellent performance in surface‐enhanced Raman Scattering for the detection of target molecules.^[61]^

Nanostructured composite materials based on diatom frustules are also represented in energy storage and conversion applications. A recent example is provided by a complex three‐dimensional composite for supercapacitor based on the mesoporous network of biosilica coated with TiO_2_ and doped with MnO_2_ nanospheres. Relevant electrical parameters were successfully improved, such as specific capacitance, cyclic stability and coulombic efficiency; a remarkable performance for such a low‐cost material based on natural diatomite.^[62]^ On a similar basis to previously mentioned examples, frustules from MPTMS‐fed diatoms bearing captured Ag^+^ ions have also been used for the fabrication of anodic materials. Such bio‐based electrodes demonstrated good overall charge‐discharge performance (Figure 4e), making them a promising source for the fabrication of electrode materials with improved electrochemical performance.^[63]^ Such modifications have the potential to improve already existing diatom‐based biophotovoltaic platforms.^[64]^

Diatom frustules have been recently introduced into the H_2_ production and storage applications, due to their special physico‐chemical characteristics (Figure 4d).^[65]^ The capacity of natural diatomite to store hydrogen has been calculated to a modest ~0.46 wt.%.^[66]^ However, it be greatly increased through functionalization; some remarkable examples include a porous carbon composite diatom skeleton material that can be easily synthetized in large quantities;^[67]^ and a transition metal alloy‐decorated diatom frustule‐graphene nanomaterial (PdCl_2_ and CoCl_2_), that has demonstrated a remarkable capacity for hydrogen storage (~4.83 wt.%) fairly close to international target values (~5.5 wt.%).^[68]^ Moreover, diatomite has been used as solid support for a nickel NP catalyst, that has been grafted onto DE's surface via a de‐sorption method. The resulting composite showed promising production of H_2_ via dry reforming of methane.^[21]^

## 4 Summary and Outlook

Diatoms are remarkable candidates for both *in vitro* and *in vivo* functionalization through various methodologies. Their biosilica frustules can be used as foundation to incorporate a myriad of different elements, from soluble salts, molecules, biomacromolecules, polymers and nanoparticles, yielding complex nanostructured materials. The collection of different approaches to frustule functionalization are growing nowadays more than ever. Every newly developed strategy for biosilica modification is determined by the nature of the functional moiety to be inserted, and the desired extent and durability of the modification.

The origin of the starting material strongly influences the practical mechanisms available for chemical modification. Very frequently DE has been chosen due to its economic affordability and wide availability, making scale up production of functionalized biosilica easier. However, the use of DE and clean PF only allows for superficial functionalization, thus limiting the load and durability of the active components. Alternatively, *in vivo* feeding allows for the bulk integration of the functional material right in the core of the diatom's shell. The number of soluble active compounds, like metallic ions, salts and organic molecules, that living diatoms can assimilate and incorporate into their frustules is continuously growing. In these terms the biocompatibility of the compounds used is essential, given that microalgae must be alive for the duration of the functionalization, for it to be operational. Thus, not only the selection of the chemical groups to use is relevant, but also their local concentration and delivery method.

The number of diatom species that are currently in use for biosilica production is relatively small, in comparison to the number of species that have been already described. The immense diversity in terms of shapes, pore patterns and sizes remain for now widely unexplored. Future efforts could focus on researching which selection of micro‐ and nanostructures fit best to specific applications and device design.

Doped biosilica fills a relevant niche in several applicative fields like bone tissue scaffolding, drug delivery and in the production of photonic and fluorescent nanocomposites. Interestingly, we are only starting to see how far they can reach in more innovative dimensions such as solid support in batteries, photovoltaic devices, supercapacitors and even catalysts for energy conversion. Special attention should be given to the potential of functionalized, living diatoms for bioremediation, since the combination of diatoms’ natural capacity to absorb pollutants and an active coating to degrade them, can make for very effective and yet biodegradable decontaminating agents; and for energy conversion, for instance in biophotovoltaics, an incipient field that has just taken off.

The use of diatom biosilica as inspiration for the synthesis and design of complex architectures, either as imitation or as straightforward mould, can provide new tailored active composites, useful in applications like biosensing and optoelectronics.

The examples herein reported demonstrate that bioinspired and biomimetic materials can be obtained by creatively altering the architecture and functional components of diatoms’ frustules, with many new approaches yet to discover. After ten years, we still think that the field of biohybrid materials, and particularly the concept of living material, is still in its infancy, henceforth, we can envisage very exciting developments in the ten years to come.

## Conflict of Interests

The authors declare no conflict of interest.

5

## Biographical Information

*Gianluca Maria Farinola is Full Professor of Organic Chemistry at the Chemistry Department of the University of Bari “Aldo Moro”. He is currently the President of the Società Chimica Italiana (2023–2025), and a Chemistry Europe Fellow. He has served as the President of the Organic Chemistry Division of EuChemS (2018–2021). His research interests are the sustainable organic synthesis of organic molecules and polymers, useful for organic optoelectronics and solar energy conversion; and the development of hybrid functional nanomaterials based on organic molecules and photosynthetic microorganisms, for applications in bio‐optoelectronics, photoconversion and biomedicine*.

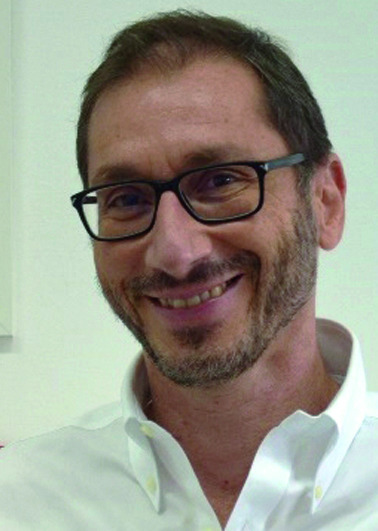

## Biographical Information

*Cesar Vicente‐Garcia obtained his M.S. in Organic Chemistry in 2019 at the Autonomous University of Madrid, and his Ph.D. degree in Chemistry and Molecular Sciences in 2024 at the University of Bari “Aldo Moro”, framed within the ITN Network “Bioinspired and Bionic Materials for Enhanced Photosynthesis”. Currently a postdoctoral fellow in the Farinola group, he works on the functionalization of living diatom microalgae and their isolated biosilica frustules to confer them novel and enhanced functions. Having a mixed chemical/biochemical background, his current research interests include the development of hybrid biomaterials based on living cells, for applications in bioremediation and bioelectronics*.

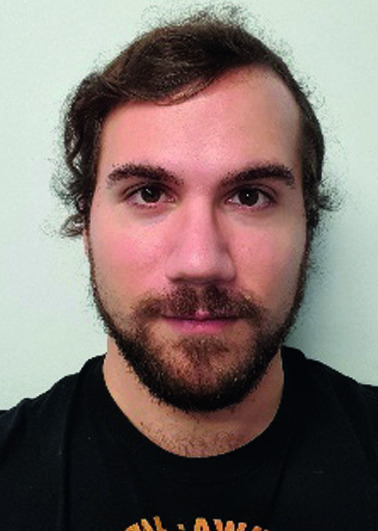

## Biographical Information

*Danilo Vona received his Ph.D. degree in Chemical Sciences in 2015 and he is currently an assistant professor in organic chemistry at the University of Bari “Aldo Moro.” His research activity focuses on bioorganic approaches to functionalize microorganisms, keeping them alive and boosting their intrinsic properties. He was the principal investigator of the project AlgAmbiente, regarding the remediation of pollutants based on modified magnetic microalgae and has been a visiting researcher in Norway and the Czech Republic*.

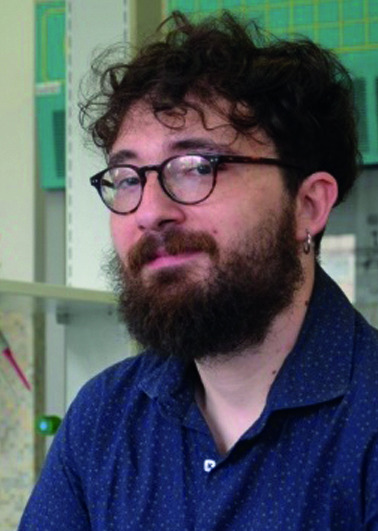

## Biographical Information

*Annarita Flemma obtained her B.S. in Biological Sciences from the University of Bari and her M.S. in Environmental Science from the University of Lecce in 2021, where she focused on developing an electrochemical sensor based on poly‐metalloporphyrins for environmental applications. She earned a research scholarship at the University of Lecce in the Environmental Analytical Chemistry laboratory as part of the INTERREG CASCADE Italy‐Croatia project. She is currently pursuing a Ph.D. in Chemical and Molecular Sciences at the University of Bari. Her research involves utilising micro‐ and macroalgae for bioremediation and developing new materials for pharmaceutical and agricultural applications*.

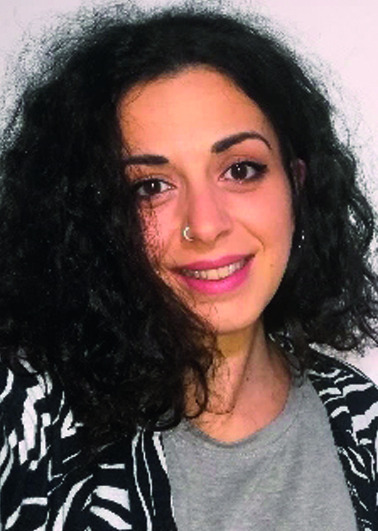

## Biographical Information

*Stefania R. Cicco graduated in chemistry in 1994 and received her Ph.D. degree in chemical sciences at the University of Bari, Italy, in 1998. After a stay at the Politecnico of Bari working on the synthesis of organometallic catalysts, she became, in 2002, a CNR researcher at the Institute of Organometallic Compounds (ICCOM). Her current research interests include the design and synthesis of organic semiconductors and the chemical modification of bioinspired materials for applications in electronics and nanobiotechnology*.

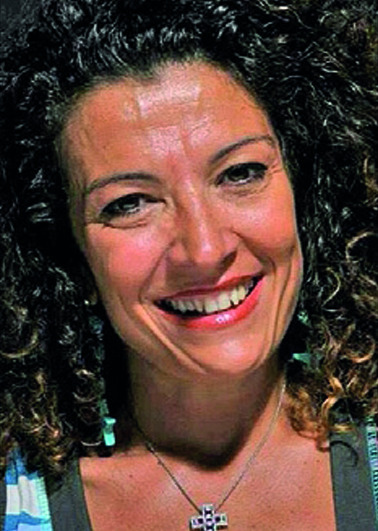

## Data Availability

Data sharing is not applicable to this article as no new data were created or analyzed in this study.
